# Oncostatin M Induces Lipolysis and Suppresses Insulin Response in 3T3-L1 Adipocytes

**DOI:** 10.3390/ijms23094689

**Published:** 2022-04-23

**Authors:** Jennifer L. Bailey, Hardy Hang, Anik Boudreau, Carrie M. Elks

**Affiliations:** 1 Matrix Biology Laboratory, Pennington Biomedical Research Center, Baton Rouge, LA 70808, USA; jennifer.bailey@pbrc.edu; 2 Adipocyte Biology Laboratory, Pennington Biomedical Research Center, Baton Rouge, LA 70808, USA; hardyhang@gmail.com (H.H.); anik.boudreau@pbrc.edu (A.B.); 3 Health Sciences Center (LSUHSC), School of Medicine, Louisiana State University, New Orleans, LA 70112, USA

**Keywords:** oncostatin M, adipocyte, lipolysis, insulin resistance, oncostatin M receptor

## Abstract

Oncostatin M (OSM) is an immune cell-derived cytokine that is upregulated in adipose tissue in obesity. Upon binding its receptor (OSMR), OSM induces the phosphorylation of the p66 subunit of Src homology 2 domain-containing transforming protein 1 (SHC1), called p66Shc, and activates the extracellular signal-related kinase (ERK) pathway. Mice with adipocyte-specific OSMR deletion (*Osmr^FKO^*) are insulin resistant and exhibit adipose tissue inflammation, suggesting that intact adipocyte OSM–OSMR signaling is necessary for maintaining adipose tissue health. How OSM affects specific adipocyte functions is still unclear. Here, we examined the effects of OSM on adipocyte lipolysis. We treated 3T3-L1 adipocytes with OSM, insulin, and/or inhibitors of SHC1 and ERK and measured glycerol release. We also measured phosphorylation of p66Shc, ERK, and insulin receptor substrate-1 (IRS1) and the expression of lipolysis-associated genes in OSM-exposed 3T3-L1 adipocytes and primary adipocytes from control and *Osmr^FKO^* mice. We found that OSM induces adipocyte lipolysis via a p66Shc-ERK pathway and inhibits the suppression of lipolysis by insulin. Further, OSM induces phosphorylation of inhibitory IRS1 residues. We conclude that OSM is a stimulator of lipolysis and inhibits adipocyte insulin response. Future studies will determine how these roles of OSM affect adipose tissue function in health and disease.

## 1. Introduction

The primary function of adipose tissue is to store energy in the form of lipid, for later use by the body. Adipocytes mobilize lipids as free fatty acids (FFA) in settings where energy needs increase, such as fasting or exercise [1,2,3]. The process by which intracellular triglycerides are hydrolyzed to FFA and glycerol, termed lipolysis, is tightly regulated at the nutritional and hormonal levels, and loss of this regulation may contribute to obesity and diabetes. Specifically, a dysregulation of lipolysis inhibition—resulting in excess FFA release even when not nutritionally required—has been implicated [1,2,3,4,5].

Many factors can stimulate lipolysis; adipose tissue cytokines, produced by immune cells, are major regulators of this process. The most well studied lipolytic cytokine in adipose tissue is tumor necrosis factor-alpha (TNFα), which exerts its lipolytic effects via several mechanisms [1,4,6,7]. The primary source of TNFα in adipose tissue is the macrophage [6]. In addition to TNFα, several other proinflammatory cytokines are stimulators of lipolysis [5,6,7,8,9,10,11].

Oncostatin M (OSM) is a member of the glycoprotein 130 (gp130)/interleukin-6 (IL6) family of cytokines which use the gp130 subunit in their receptor complexes [12,13]. The effects of OSM can be anti- or pro-inflammatory depending on cell type and physiological context [12,13]. We and others have reported that OSM signaling in adipocytes induces the expression of pro-inflammatory mediators and that adipose tissue OSM expression increases in human and murine obesity [14,15,16]. OSM is unique among the IL6 cytokines in that it has its own specific receptor (oncostatin M receptor; OSMR) that mediates its effects [17] via Janus kinase (JAK)-signal transducer and activator of transcription (STAT) or extracellular signal-regulated kinase (ERK) pathway activation [18,19,20,21,22].

A distinguishing feature of OSM signaling is the recruitment of the p66 isoform of the adaptor protein, Src homology 2 domain-containing transforming protein 1 (SHC1), referred to as p66Shc, to tyrosine 861 (Y861) of OSMR [21,22,23]. This non-redundant signaling mechanism of OSMR is not attributed to any other gp130 cytokine [23]. Phosphorylation of Y861 allows direct binding of p66Shc to OSMR and induces p66Shc phosphorylation and the activation of ERK1/2 [21,22]. While other gp130 receptors activate ERK1/2 via an Src homology region 2 domain-containing phosphatase-2 (SHP2)-dependent mechanism, OSMR lacks the SHP2 recruitment motif and uses p66Shc activation as its mode of ERK1/2 activation [22,23].

Our previous work in a mixed-background mouse model has demonstrated that mice lacking OSM signaling specifically in adipocytes (*Osmr^FKO^* mice) are insulin-resistant and have increased adipose tissue inflammation when compared to control mice (*Osmr^fl/fl^*) [14,24]. While OSM signaling has a clear role in adipose tissue function, the precise effects of OSM on the adipocyte itself remain unclear. In this study, we assessed the effects of OSM on adipocyte lipolysis and insulin sensitivity. We found that OSM stimulates lipolysis through the p66Shc-ERK pathway and inhibits the adipocyte response to insulin via phosphorylation of inhibitory residues of IRS1.

## 2. Results

### 2.1. OSM Stimulates Adipocyte Lipolysis and Diminishes the Inhibitory Effects of Insulin on Lipolysis

**Figure 1:**
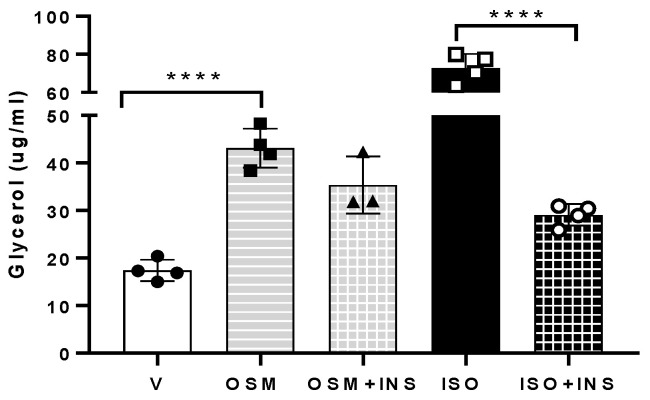
OSM induces lipolysis and partially inhibits its suppression by insulin. Lipolysis assays were conducted in fully differentiated 3T3-L1 adipocytes. Cells were pretreated with OSM overnight and, the following morning, were incubated in medium containing phosphate buffered saline (PBS) + 0.1% bovine serum albumin (BSA) as the vehicle control (V), 0.5 nM OSM, 0.5 nM OSM + 17.5 nM bovine insulin (INS), 2 nM isoproterenol (ISO) as a positive control, or ISO + INS (as a positive control for insulin suppression), for 6 h. Glycerol measurements were obtained from 50 μL media from each well (n = 3–4 wells per condition). OSM robustly stimulated lipolysis. This effect was maintained in the presence of insulin, suggesting that OSM inhibits the insulin-induced suppression of lipolysis. This experiment was conducted twice on separate batches of adipocytes. Significant differences are denoted as follows: **** *p* < 0.0001 between groups; bars represent mean ± SEM.

Increased adipocyte ERK activation was identified as a contributor to the increased basal lipolysis observed in obesity [5]. This finding, along with the established role of OSM in activating the ERK pathway [22,25,26,27], prompted us to examine how OSM affected adipocyte lipolysis. When adipocytes were exposed to OSM, glycerol release significantly increased (Figure 1). When OSM-exposed adipocytes were also exposed to insulin, glycerol release remained elevated (Figure 1), in contrast to lipolysis induced by the nonselective beta-adrenergic agonist isoproterenol, which was almost completely abrogated by insulin. Taken together, these results suggest that: (a) OSM induces adipocyte lipolysis and (b) OSM inhibits the insulin suppression of lipolysis.

### 2.2. Inhibitory Phosphorylation of Insulin Receptor Substrate 1 (IRS1) Is Significantly Increased in OSM-Exposed Adipocytes

**Figure 2:**
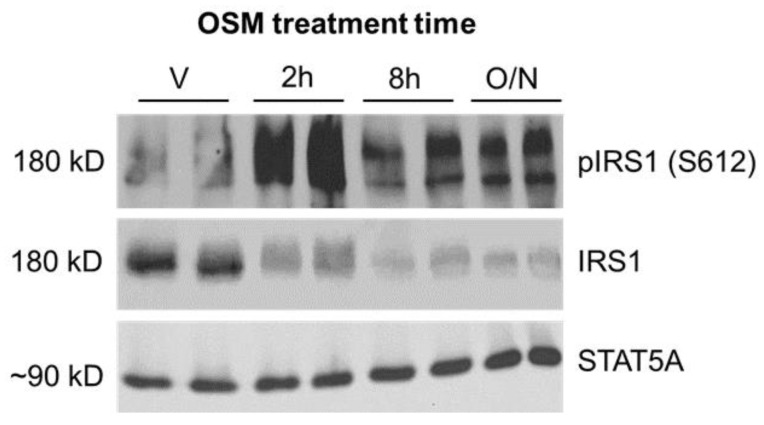
OSM induces inhibitory phosphorylation of IRS1 at serine 612 (S612). 3T3-L1 adipocytes were exposed to 0.5 nM OSM for 2 h, 8 h, or overnight (~16 h). Cells were harvested after OSM exposure, and lysates were subjected to immunoblotting for phospho-IRS1 (S612) and total IRS1; STAT5A was used as a loading control. OSM stimulated inhibitory IRS1 phosphorylation of S612 at all time points depicted. The immunoblot pictured here represents results from two independent experiments, each with 3 wells per condition (n = 6 total wells per condition).

Insulin sensitivity is regulated in part by serine and threonine phosphorylation of the IRS1 and IRS2 proteins [28,29]. Specific phosphorylation events can activate or inhibit IRS proteins, depending on the specific residues involved. Phosphorylation of human serine (S) 616/mouse S612 results in the inhibition of IRS function [28,29]. One kinase mediating S612 phosphorylation is ERK1/2 [28,29]. As OSM is a known ERK1/2 activator and insulin suppression of lipolysis is inhibited in OSM-exposed adipocytes, we assessed IRS1 phosphorylation at S612 as a possible mechanism. Indeed, a sustained increase in S612 phosphorylation was observed in adipocytes exposed to OSM (Figure 2) when compared to vehicle-treated adipocytes. These results suggest that OSM increases inhibitory serine phosphorylation of IRS1 and that IRS1 inhibition may contribute to OSM’s ability to prevent insulin-induced suppression of adipocyte lipolysis.

### 2.3. OSM Induces p66Shc Phosphorylation and ERK 1/2 Activation in Adipocytes

**Figure 3:**
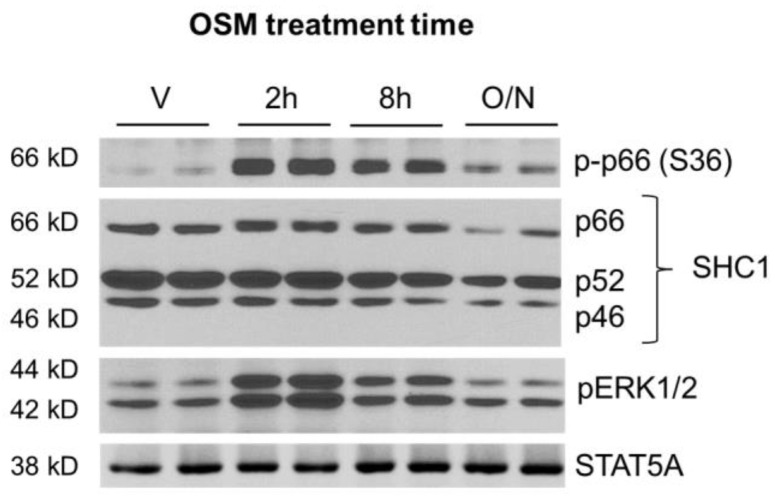
OSM induces phosphorylation of the p66 isoform of SHC1 at serine 36 (S36). 3T3-L1 adipocytes were exposed to 0.5 nM OSM at various times. Cells were harvested after OSM exposure and subjected to immunoblotting for phospho-p66 (S36), total SHC1, and phospho-ERK1/2; STAT5A was used as a loading control. OSM strongly stimulated p66Shc and ERK1/2 phosphorylation at 2 h and 8 h of treatment. The immunoblot depicted here represents results from two independent experiments, each with 3 wells per condition (n = 6 total wells per condition).

The phenomenon of OSM binding to promote p66Shc recruitment and subsequent ERK 1/2 activation has not been described in the adipocyte. We first assessed whether OSM promoted p66Shc phosphorylation in adipocytes, using the same cell lysates used to measure OSM-mediated IRS1 phosphorylation (described in Section 2.2 and Figure 2). In addition to assessing p66Shc phosphorylation, we also assessed SHC1 abundance and ERK1/2 phosphorylation. Both p66Shc and ERK1/2 were robustly phosphorylated by OSM at 2 and 8 h post-exposure; these effects were decreased in adipocytes exposed to OSM overnight (Figure 3).

### 2.4. SHC1 Inhibition Abrogates OSM-Induced Adipocyte Lipolysis

**Figure 4:**
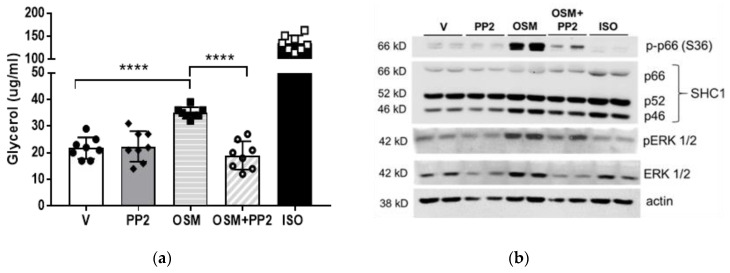
Pharmacological inhibition of SHC1 abrogates OSM-stimulated adipocyte lipolysis. (**a**) Fully differentiated 3T3-L1 adipocytes were pretreated with the SHC1 inhibitor, PP2 (10 μM), in a regular maintenance medium the night before the assay. On the day of the assay, adipocytes were placed into an incubation medium containing V, the SHC1 inhibitor PP2 (10 μM), 0.5 nM OSM, OSM + PP2, or 10 μM ISO as a positive control, for 4 h. Glycerol measurements were obtained from 50 μL media (n = 7–8 wells per condition). PP2 alone did not affect basal lipolysis, but completely inhibited the OSM induction of lipolysis. (**b**) Immunoblotting of cell lysates from (**a**) was used to verify SHC1 inhibition using p66Shc and ERK phosphorylation as readouts. This experiment was conducted twice on independent batches of adipocytes. Significant differences are denoted as follows: **** *p* < 0.0001 between groups.

Since OSM promotes the phosphorylation of both p66Shc and ERK1/2 in adipocytes (Figure 3), we next assessed the role of SHC1 in OSM-stimulated lipolysis using PP2, a pharmacological inhibitor of SHC1. No obvious effect of SHC1 inhibition on basal lipolysis was observed (Figure 4a), but a significant decrease in OSM-stimulated glycerol release was observed in cells pre-treated with PP2 (Figure 4a). Immunoblotting of cell lysates from the same experiment was used to verify SHC1 inhibition using p66Shc phosphorylation as a readout. We observed significant increases in p66Shc and ERK phosphorylation by OSM, which were inhibited by PP2 (Figure 4b).

### 2.5. Mitogen-Activated Protein Kinase-Kinase (MEK) or OSMR Blockade Inhibits OSM-Induced Adipocyte Lipolysis

**Figure 5:**
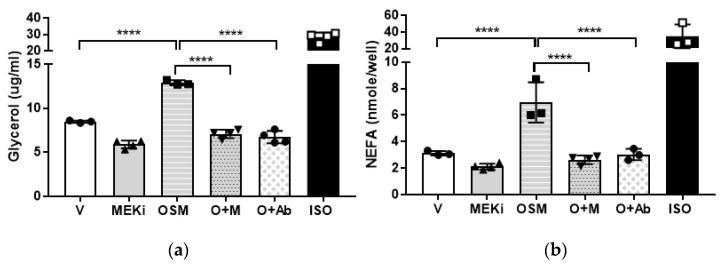
Pharmacological inhibition of MEK or antibody inhibition of OSMR abrogates OSM-stimulated adipocyte lipolysis. Fully differentiated 3T3-L1 adipocytes were pretreated with OSM in lipolysis incubation medium the night before the assay. On the day of the assay, adipocytes were incubated with or without the acyl-CoA synthetase inhibitor, triacsin C (for NEFA and glycerol measurements, respectively), plus V, the MEK inhibitor (MEKi) U0126 (50 mM), 0.5 nM OSM, OSM + U0126 (O + M), OSM + 10 μg of anti-OSMR antibody (O + Ab), or 10 μM ISO as a positive control, for 4 h. (**a**) Glycerol measurements were obtained from 50 μL media without triacsin C (n = 3–4 wells per condition). (**b**) NEFA measurements were obtained from 50 μL media containing triacsin C (n = 3–4 wells per condition). MEK inhibition or the addition of anti-OSMR antibody completely inhibited OSM-induced glycerol and NEFA release. This experiment was conducted in duplicate on a single batch of adipocytes. Significant differences are denoted as follows: **** *p* < 0.0001 between groups.

We then examined the roles of OSMR and the ERK pathway in OSM-induced lipolysis. A cascade of events must occur upstream of ERK before it is phosphorylated and activated, with the most proximal event being the activation of MEK [30,31,32]. MEK directly phosphorylates ERK to render it active [30,31], and is a major point of signal amplification in the Ras-MEK-ERK cascade [30,31,32]. Assays were performed using the MEK inhibitor, U0126, and an anti-OSMR antibody; both glycerol and FFA release from adipocytes were measured. Consistent with previous reports, inhibition of the ERK pathway decreased basal lipolysis [5]. When U0126- or anti-OSMR antibody-treated adipocytes were exposed to OSM, both glycerol and FFA release (Figure 5a,b, respectively) remained at basal levels. These findings suggest that OSMR is necessary for the OSM-induced lipolysis that occurs via a mechanism involving p66Shc and ERK.

### 2.6. OSM Exposure and OSMR Deletion Have Opposing Effects on Lipolysis-Associated Genes in Adipocytes

**Figure 6:**
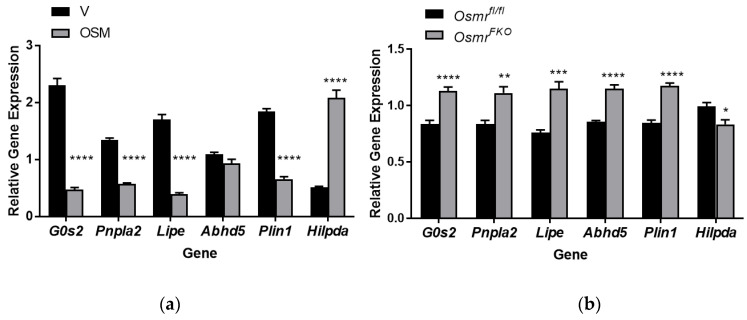
OSM exposure and OSMR loss differentially affect the expression of genes associated with lipolysis. (**a**) Fully differentiated 3T3-L1 adipocytes were exposed to 0.5 nM OSM in lipolysis incubation medium for 4 h. Cells were harvested for RNA synthesis and quantitative RT-PCR was performed for the genes indicated. (**b**) Primary adipocytes isolated from Osmr^fl/fl^ and Osmr^FKO^ mice were fully differentiated and then harvested for RNA synthesis. Quantitative RT-PCR was performed for the genes indicated. This experiment was conducted in duplicate on a single batch of adipocytes. Significant differences are denoted as follows: * *p* < 0.1, ** *p* < 0.01, *** *p* < 0.001, **** *p* < 0.0001 between groups.

The regulation of lipolysis is complex and is influenced by transcriptional events in addition to post-translational modifications [1,2,3]. As a first step toward understanding how OSM may affect the transcription of lipolysis-associated genes, we analyzed RNA from 3T3-L1 adipocytes treated with OSM for 4 h and from OSMR-null or control adipocytes obtained from our *Osmr^FKO^* and *Osmr^fl/fl^* mice, respectively. We analyzed the expression of several key genes including *Pnpla2* (encodes adipose triglyceride lipase (ATGL)), *Lipe* (encodes hormone-specific lipase (HSL)), *G0s2* (inhibitor of ATGL activity), *Abhd5* (encodes comparative gene identification-58 (CGI-58), an ATGL activator), *Plin1* (encodes perilipin 1), and *Hilpda* (hypoxia-induced lipid droplet associated protein). OSM significantly decreased the expression of *G0s2*, *Pnpla2*, *Lipe*, and *Plin1*, while significantly increasing *Hilpda* expression; *Abhd5* expression was not affected by OSM treatment (Figure 6a). In OSMR-null cells, the opposite effects were observed—expression levels of *G0s2*, *Pnpla2*, *Lipe*, *Abhd5*, and *Plin1* were significantly higher, while *Hilpda* expression was significantly lower (Figure 6b). These results suggest that exposure of adipocytes to OSM leads to diminished expression of lipolysis-associated genes. In addition, by significantly increasing gene transcription, the loss of OSMR may serve to sensitize the adipocyte to other lipolysis-inducers since the OSM response is absent. Further investigation is required to determine how these gene expression differences relate to protein expression levels.

## 3. Discussion

Adipocyte lipolysis is crucial to providing needed substrates to peripheral tissues in conditions of low energy availability or high demand (fasting or exercise, for instance). Conversely, insulin signaling induced by feeding inhibits lipolysis and enhances lipogenesis. Obese and insulin-resistant states are associated with elevated rates of basal lipolysis in the fed state, due in part to impaired insulin action in adipose tissue [4]. Chronically high levels of circulating FFAs, in turn, exacerbate metabolic dysfunction, and pharmacological inhibition of lipolysis improves insulin sensitivity and glucose tolerance in subjects with obesity and/or type 2 diabetes and in mouse models of diet-induced obesity [33,34,35,36].

Immune cell-derived cytokines can positively or negatively influence adipocyte function and, therefore, the overall function of adipose tissue. Adipose tissue inflammation, a hallmark of obesity and insulin resistance, contributes to aberrant lipolysis in an insulin-independent manner, particularly through the actions of TNFα and other inflammatory cytokines driving obesity-related adipose tissue inflammation [5,6,7,8,9,10,11]. Our previous work demonstrates that adipocytes respond to OSM by producing inflammatory mediators [14]. We have also reported that OSM can suppress the gene expression and secretion of stromal-derived factor 1 (SDF1), a chemokine that has been implicated in diet-induced obesity, in adipocytes [15]. How these functions of OSM converge to produce physiological outcomes remains unclear. Also unclear is how OSM may directly influence lipid handling in the adipocyte. As a first step toward understanding the relationship between OSM and adipocyte lipid balance, we assessed the effects of OSM on lipolysis. We demonstrate for the first time here that OSM induces both adipocyte lipolysis and inhibitory IRS1 serine phosphorylation and that OSMR loss induces the expression of lipolysis-associated genes in adipocytes.

The data presented here clearly demonstrate that OSM acts on adipocytes to: (1) stimulate lipolysis, (2) inhibit IRS1 phosphorylation, (3) stimulate p66Shc phosphorylation, and (4) induce ERK1/2 phosphorylation. Our findings using either antibody inhibition of OSMR or a MEK inhibitor suggest that OSM-induced lipolysis occurs via a p66Shc-ERK-OSMR mechanism. These findings are in line with the known ERK activating effects of OSM in other cell types [19,21]. Our studies provide new insight on the direct effects of OSM on adipocyte function, but some limitations to these studies do exist. First, OSM also activates the JAK-STAT pathway [18,20], though we did not examine this pathway in the current studies. We and others have demonstrated that both STAT5 and STAT3 are critical mediators of adipocyte lipid metabolism [37,38], so we cannot exclude the possibility that OSM could also influence lipolysis via STAT signaling.

Second, we have clearly demonstrated here that OSM induces p66Shc and ERK1/2 phosphorylation and that these events occur over similar times (Figure 3 and Figure 4b). Our findings imply that p66Shc phosphorylation may precede ERK1/2 phosphorylation in our model, but we have not conclusively demonstrated this effect. Our future studies will include adipocytes with inactivating mutations in the OSMR Y861 docking site for p66Shc. Using these OSMR mutant adipocytes, we can determine the true dependence of ERK1/2 activation on p66Shc phosphorylation. SHC1 is involved in a number of signaling cascades, so it is also possible that ERK1/2 activation is not the only lipolysis-promoting signaling pathway affected by p66Shc.

Finally, we show here that OSM stimulates lipolysis in a manner that depends, at least in part, on OSMR, p66Shc, and ERK1/2. However, the downstream events that trigger lipolysis transcriptionally, at the post-translational level, or both, remain unclear. In line with our gene expression findings in OSM-exposed adipocytes, ERK1/2 activation can result in decreased transcription of *G0s2* [2,39,40], which leads to increased ATGL activity. In addition, ERK1/2 is reported to phosphorylate HSL at serine 600 [5], but there are no commercially available antibodies for measuring this phosphorylation event. Follow-up studies in OSM-exposed adipocytes will aim to determine the downstream mechanisms by which OSM stimulates lipolysis.

Though we are the first to describe OSM effects on lipolysis, the effects of OSM on other aspects of lipid handling and insulin response have been described in both adipose tissue and the liver [25,41,42,43,44,45]. In HepG2 cells, OSM strongly upregulates the low-density lipoprotein (LDL) receptor, increases diacylglycerol metabolism, and activates transcription of long-chain acyl-CoA synthetases 3 and 5 [25,41,42,43]. Mice with global OSMR deletion exhibit severe hepatic steatosis accompanied by increased expression of hepatic fatty acid synthesis genes and significantly higher fat mass [44,45]. The obvious question arising when our new in vitro findings and our previous in vivo findings in the *Osmr^FKO^* mouse are taken together is: if OSM induces adipocyte lipolysis—a seemingly harmful effect that promotes insulin resistance in obesity—how does adipocyte OSMR loss (and the loss of OSM-induced lipolysis) still result in adipose tissue inflammation and insulin resistance [14,15,24]?

There are several possible explanations, and all will require intensive further study. First, the autocrine action of SDF1 on adipocytes is reported to induce inhibitory IRS1 phosphorylation and result in insulin resistance in mice [46]. Given our previous observation that OSM directly suppresses the expression and secretion of adipocyte SDF1 [15], the loss of this suppressive function of OSM may cause aberrant SDF1 secretion leading to increased adipocyte insulin resistance. Second, adipocyte OSM-OSMR signaling may be required to maintain the balance between lipid storage and immune cell cytokine production. Macrophages are the primary producers of OSM in adipose tissue, and the macrophage OSM–adipocyte OSMR signal may be important in signaling the release of lipid from the adipocyte for use by the macrophage. In support of this possibility, macrophages with an anti-inflammatory phenotype may rely more heavily on fatty acid metabolism than inflammatory macrophages [47,48,49]. Could an OSM–OSMR-induced lipid signal be required to maintain macrophage function? Third, preadipocytes express OSMR [27], so if intact adipocyte OSMR signaling is absent, could excess OSM signaling on preadipocytes inhibit adipogenesis and promote inflammatory mediator production by the preadipocyte? We have observed increased OSM expression in adipose tissue of *Osmr^FKO^* mice [14], so enhanced paracrine OSM signaling in this setting may be a reasonable suggestion.

Further research regarding OSM and adipocyte lipid metabolism is required, but our present findings identifying OSM as a novel contributor to adipose tissue lipolysis provide valuable insight into the relationship between elevated lipolysis and insulin resistance.

## 4. Materials and Methods

### 4.1. 3T3-L1 Adipocyte Culture and OSM Exposure Time Course

Murine 3T3-L1 preadipocytes were grown to two days post-confluence and differentiated into adipocytes as previously described [14,15]. Seven to ten days after fully differentiated, cells were exposed to vehicle (0.1% BSA in PBS) or 0.5 nM recombinant murine OSM (R&D Systems, Minneapolis, MN, USA; Catalog #495-MO-025) for 2 h, 8 h, or overnight (~16 h), then harvested for immunoblotting (Figure 2 and Figure 3) as described below. Additional batches of cells were assayed and treated as described in 4.2 below.

### 4.2. Glycerol and NEFA Assays

Lipolysis assays were conducted on adipocytes grown in 12-well plates. Adipocytes were placed in an incubation medium with or without triacsin C (containing phenol red-free DMEM, low glucose, and fatty acid-free BSA, and allowed to rest for one hour. Cells were then exposed to conditions as described here for each experiment. For the experiment in Figure 1, vehicle, OSM (0.5 nM), isoproterenol (ISO, positive control; 2nM; Sigma-Aldrich, St. Louis, MO, USA; #I-5752), or bovine insulin (17.5 nM; Sigma-Aldrich; I-5500) were used. For the experiment in Figure 4, vehicle, OSM, the SHC1 inhibitor PP2 (10μM; Selleck Chemicals, Houston, TX, USA; #S7008), or 10 nM ISO were used. For the experiment in Figure 5, vehicle, OSM, triacsin C (5 μM; Cayman Chemical, Ann Arbor, MI, USA; # 10007448), the MEK inhibitor, U0126 (50mM; Cayman Chemical, #70970), or an anti-OSMR antibody (10 μg/well; Santa Cruz Biotechnology, Dallas, TX, USA; #sc-8493), were used. Adipocytes were exposed to conditions as noted in figure legends, for 4–6 h. Media was collected from each well and assayed for glycerol content using free glycerol reagent and glycerol standard (Sigma-Aldrich, #F6428 and #G7793, respectively). NEFA release into triacsin C-free culture media was quantified using a Free Fatty Acid Kit (Sigma-Aldrich, #MAK044) according to the manufacturer’s instructions.

### 4.3. Immunoblotting

Protein concentrations of cell lysates were quantified, 50–1000 µg protein per well loaded on 5% or 10% polyacrylamide gels, and gels transferred to 0.45 µm polyvinylidene fluoride membranes (ThermoFisher, Waltham, MA, USA; #88518) as described previously [14,15,24]. Membranes were probed and imaged using standard immunoblotting techniques. Proteins were detected using 1:10,000 dilutions of species-appropriate horseradish peroxidase-conjugated secondary antibodies (Jackson ImmunoResearch, West Grove, PA, USA) in 1% non-fat dry milk (NFDM) and SuperSignal West Pico PLUS reagents (ThermoFisher). Primary antibodies and diluents used for immunoblotting appear in Table 1 below.

### 4.4. Primary Adipocyte Culture from Osmr^fl/fl^ and Osmr^FKO^ Mice

Isolation and culture of murine adipose-derived stem cells was performed using methods described in [50]. Briefly, male *Osmr^fl/fl^* and *Osmr^FKO^* mice (n = 4 per genotype; 4 weeks of age) were humanely euthanized and their fur was thoroughly moistened with 70% ethanol. Inguinal adipose tissue was quickly excised from each animal and placed into warm antibiotic-containing Hank’s balanced salt solution (HBSS). Under a laminar flow hood, tissues were pooled by genotype. Each pool was rinsed well in warmed HBSS + antibiotics then transferred to collagenase digestion buffer (1 mg/mL type I collagenase in HBSS supplemented with DNase I and magnesium chloride) [50] and minced finely.

Minced tissues were placed into sterile 50 mL conical tubes and collagenase buffer volume increased to 10 mL. Tubes were placed into a 37 °C shaking water bath at 200 rpm until dissociated (approximately 1.5 h). Once dissociated, each tissue suspension was filtered through a sterile 100 μm filter into a fresh 50 mL tube and centrifuged for 5 min at 450× *g* at room temperature to separate the primary adipocytes and stromal vascular cell (SVC) pellet. The floating adipocytes and supernatant were aspirated and the SVC pellet was resuspended in 2 mL ammonium-chloride-potassium (ACK) lysis buffer, triturated 5–8 times, and incubated at room temperature for 3 min. 10 mL Dulbecco’s phosphate-buffered saline (DPBS) was added to neutralize the ACK lysis buffer, cells were centrifuged at 450× *g* at room temperature for 5 min and the supernatant aspirated. The pellet was then resuspended in 2 mL of stromal medium [50] per gram of adipose tissue and counted; cells were then plated at a density of ~160,000 cells per well of a 6-well plate (2 wells per genotype). Cells were incubated at 37 °C with 5% CO_2_ for 24 h, rinsed, and placed in fresh stromal media.

Two days after plating, cells were trypsinized and plated in 12-well plates at a density of ~7000 cells per well (five 12-well plates per genotype), and stromal media changed every other day. Five days after cells were placed into 12-well plates, at approximately 90% confluence, medium was changed to differentiation medium [50]. Three days post-differentiation, cells were placed into maintenance medium [50]. Once cells were fully differentiated (day 10 post-differentiation), they were harvested for RNA extraction and later gene expression analyses.

### 4.5. Gene Expression Analyses

Total RNA was isolated from cell monolayers using the RNeasy Mini Kit (Qiagen, Germantown, MD, USA) as previously described [14,15,24]. RNA concentrations were quantified and reverse transcription performed with 2 µg of RNA as previously described [15]. Quantitative PCR was then performed using previously described materials and thermal cycling conditions [15], and all target genes were normalized to *Ppia* (peptidyl prolyl isomerase A). Primers used were from Integrated DNA Technologies (Coralville, IA, USA); primer sequences appear in Table 2.

## 5. Conclusions

In these studies, we demonstrate for the first time that OSM stimulates lipolysis and increases inhibitory IRS1 phosphorylation in cultured adipocytes. Our data suggest that OSM-stimulated lipolysis is OSMR-dependent and occurs via a p66Shc-ERK1/2-mediated mechanism. We also show that adipocyte-specific loss of OSMR leads to upregulated expression of several key genes involved in lipolysis. We conclude from these results that OSM is a lipolysis inducer in vitro and deserves further study in the context of obesity and metabolic disease.

## Figures and Tables

**Table 1:** Primary antibodies used for immunoblotting.

Antibody	Manufacturer	Catalog Number	Dilution (Diluent)
anti-IRS1	Cell Signaling ^1^	3194	1:1000 (5% NFDM)
anti-pIRS1 (S612)	Cell Signaling	2386	1:1000 (5% BSA-TBST) ^2^
anti-STAT5A	Abcam ^3^	ab32043	1:1000 (1% BSA- TBST)
anti-p-p66 (S36)	Millipore Sigma	566807	1:500 (1% BSA-TBST)
anti-Shc1	R&D Systems	MAB7129	1:500 (1% BSA-TBST)
anti-pERK1/2	Promega ^4^	V8031	1:5000 (1% BSA-TBST)
anti-ERK 1/2	Cell Signaling	4695	1:1000 (1% BSA-TBST)
anti-β actin	Santa Cruz	sc-47778	1:1000 (1% BSA-TBST)

^1^ Danvers, MA, USA. ^2^ TBST = tris-buffered saline + 0.1% Tween 20. ^3^ Cambridge, MA, USA. ^4^ Madison, WI, USA.

**Table 2:** Primers used for qPCR.

Gene	Forward Sequence	Reverse Sequence
*G0s2*	CCT GCA CAC TTT CCA TCT GA	CAA AGC CAG TCT GAC GCA A
*Pnpla2*	CTC ATA AAG TGG CAA GTT GTC TG	GAG CTC ATC CAG GCC AAT
*Lipe*	CTG CAA GAG TAT GTC ACG CTA	CTC GTT GCG TTT GTA GTG C
*Abhd5*	CCC ACA TCT ACA TCA CAC CTT	GAG AGA ACA TCA GCG TCC ATA
*Plin1*	CGT GGA GAG TAA GGA TGT CAA TG	GTG CTG TTG TAG GTC TTC TGG
*Hilpda*	TTC CAC GTT GCG ACT CAG	GAA GTA GAG CTG CTT TCT GCT
*Ppia*	CCA CTG TCG CTT TTC GCC GC	TGC AAA CAG CTC GAA GGA GAC GC

## Data Availability

The data presented in this study are available on request from the corresponding author.
